# Daily Glucocorticoid Replacement Dose in Adrenal Insufficiency, a Mini Review

**DOI:** 10.3389/fendo.2022.897211

**Published:** 2022-06-29

**Authors:** Celina M. Caetano, Carl D. Malchoff

**Affiliations:** Division of Endocrinology and Metabolism, and Neag Comprehensive Cancer Center, University of Connecticut (UCONN) Health, Farmington, CT, United States

**Keywords:** adrenal insufficiency, glucocorticoid, Hydrocortisone, replacement, guidelines, cortisol

## Abstract

The Endocrine Society Guidelines and recent reviews of adrenal insufficiency (AI) recommend a daily glucocorticoid replacement dose of 15 to 25 mg with a midpoint of 20 mg of hydrocortisone (HC) (alternatively 3 to 5 mg prednisolone) in divided doses in otherwise healthy individuals with AI. In contrast, a daily glucocorticoid replacement dose of 4.3 to 26 mg/d HC with a midpoint of 15 mg/d is predicted from current measurements of daily cortisol production rates and oral HC bioavailability. The higher HC doses recommended in the current guidelines may result in glucocorticoid overtreatment of some AI patients and associated long-term adverse outcomes. A titration method for determination of the individual patient’s daily glucocorticoid replacement dose and the impact of lower doses are reviewed. Future related research questions are identified.

## Introduction

The Endocrine Society guidelines and a recent review in The Lancet both recommend 15 to 25 mg/d of hydrocortisone (HC) or its equivalent in divided doses as the daily glucocorticoid replacement dose in otherwise healthy adults with primary adrenal insufficiency (AI) (1) and with either primary or secondary AI (2). As alternatives, the Endocrine Society suggests either prednisolone (3 to 5 mg) or cortisone acetate (20 to 35 mg) in divided doses. This focused review 1) examines the assertions/assumptions of these guidelines and predicts a broader range and lower midpoint of the daily glucocorticoid replacement dose, 2) summarizes potential complications of overtreatment, 3) presents a pragmatic down titration approach for individualizing the daily glucocorticoid replacement dose, 4) reviews the outcomes of individualized doses, and 5) proposes potential new research questions.

## The Daily Glucocorticoid Replacement Dose

### Assertions and Assumptions of the Current Recommendations

Although there are multiple reviews of AI, we have chosen the nearly identical recommendations for the daily glucocorticoid replacement dose from the Endocrine Society Guidelines (1) and those from a recent The Lancet publication by Husebye, et al. (2) as representative of views across Western economies. The Endocrine Society Guidelines address only primary AI (1) while Husebye’s treatment recommendations apply to both primary and secondary AI (2). Both recommend a HC dose of 15 to 25 mg/d (1, 2). These recommendations can be derived from the following assumptions/assertions. 1) The daily glucocorticoid replacement dose equals the daily cortisol production rate (DCPR); 2) the mean DCPR is 7.0 mg/m^2^; 3) the individualized daily glucocorticoid replacement dose should reflect the range of mean DCPR from different studies (5 mg/m^2^ to 8 mg/m^2^); 4) the oral HC bioavailability is about 70 percent; and 5) the DCPR is applied to a male of average height and ideal body weight (175 cm, 73 kg, 1.9 m^2^ BSA by the Mosteller formula). The last three of these assumptions/assertions are subject to modification or challenge.

### Alternative Predictions of the Daily Glucocorticoid Replacement Dose

**Table 1** summarizes the assertions/assumptions used in the current recommendations and compares this to our interpretation of current literature. Without data to the contrary, the unproven assumption that cortisol replacement should equal production is accepted. There is general agreement that the DCPR is accurately measured by a 30-hour stable isotope dilution methodology or by deconvolution analysis, and the mean DCPR in the largest of these studies is 7.0 mg/m^2^ (3). Earlier measurements suggested that the DCPR was about 12-15 mg/m^2^, and this estimate led to daily HC replacement dose recommendations of about 20 to 30 mg (4). Estaban et al, review the methodologic errors that led to overestimates (5). Some studies used radioactive isotope dilution methodology of brief duration in the morning when cortisol production is at its highest (5, 6). As anticipated, lower DCPR were observed in studies that lasted for ≥ 24 h. Using deconvolutional analysis of serum cortisol measurements at 20 m intervals for 24 h, Kerrigan et al. reported a DCPR of 5.3 ± 0.5 mg/m^2^ in 9 Tanner stage IV and V pubertal males and 6.1 ± 0.4mg/m^2^ in 9 Tanner stage I and II pubertal males (7). Using the 31-hour stable isotope dilution/mass spectrometry methodology in a group of 12 normal volunteers (mean age = 28 y), Esteban reported a mean DCPR of 5.7 mg/m^2^ (5). Using this same technique in a larger group of adult men (n = 24) and women (n = 30) ranging from age 19 y to 70 y, Purnell, et al. observed a some DCPR of 7.0 (range = 2.7-14) mg/m^2^. A positive correlation between age and DCPR may account for this latter DCPR estimate exceeding that observed using deconvolutional analysis in pubertal males (7) and the initial measurements by Esteban, et al. (5).

**Table 1:** Comparison of Current Guidelines for treating adrenal insufficiency with our interpretation of the current literature.

	Current Guidelines	Our Interpretation of the Current Literature
**Mean daily cortisol prod. **	7.0 mg/m2	7.0 mg/m2
		
**Range daily cortisol prod.**	5.2 - 8.8 mg/m2 (est)	2.7 - 14 mg/m2
		
**HC bioavailability**	70 percent (est)	100 percent
		
**Midpoint HC replacement**	20 mg/d	100 percent
		
**Range HC replacement**	15 - 25 mg/d	4.3 - 22.3 mg/d
**Woman, av height, ibw**		
		
**Midpoint HC replacement**	20 mg/d	15.6 mg/d
**Man, av height, ibw**		
		
**Range HC replacement**	15 - 25 mg/d	5.2 - 26 mg/d
**Man, av height, ibw**		
		
**Midpoint HC replacement**	20 mg/d	15 mg/d
**All patients**		
		
**Range HC replacement**	15 - 25 mg/d	4.3 - 26 mg/d
**All patients**		

Abbreviations. Average (av), ideal body weight (ibw), our estimate/interpretation by 34 calculating from the current guidelines (est).

### Other Assertions Are Subject to Challenge

First, the guidelines recommend a daily glucocorticoid replacement dose of 15 to 25 mg. The individualized daily glucocorticoid replacement dose should be within the DCPR population range. In their large diverse population Purnell, et al. reported a 5.2-fold DCPR range (2.7 to 14 mg/m^2^) (3). This 5.2-fold range is not reflected in the 1.7 fold range of daily glucocorticoid replacement in the current recommendations (1, 2). Second, the bioavailability of oral HC likely exceeds 70 percent. An older study using a cortisol radioimmunoassay reports that the bioavailability of oral HC is about 90 percent (8), and a recent study using more accurate LC-MS/MS cortisol measurement reports that the bioavailability is 100 percent (9). Third, the current recommendations do not account for BSA diversity. By correcting these assertions and accounting for diversity by including a woman of average height and ideal body weight (163 cm, 54.4 kg, 1.6 m^2^ BSA by the Mosteller formula), then a combined male and female predicted mean daily HC replacement dose (derived from mean DCPR = 7.0 mg/m^2^) is 12 mg; the predicted range (derived from a DCPR range of 2.7 to 14 mg/m^2^) is 4.3 to 26 mg. These values vary considerably from those of current recommendations (**Table 1**). There is evidence to support the use of a BSA adjustment. Mah et al, demonstrated that serum cortisol concentrations fall into a narrower range when a BSA adjusted HC dose is compared to a fixed dose (10). Finally, the potential effect of age is not incorporated into the current guidelines (1, 2).

Based on current DCPR measurements some authors have suggested that the daily glucocorticoid replacement dose be less than the standard 15 to 25 mg HC dose or 3 to 5 mg prednisolone (11–13).

## Potential Complications of Glucocorticoid Overreplacement

Accumulating evidence suggests that mild long-term glucocorticoid excess may be harmful. In a variety of AI groups conventional daily glucocorticoid replacement doses are associated with complications of glucocorticoid excess including death from cardiometabolic disorders and infections (14–16). These and evidence of other potential complications have been carefully reviewed (12). Similarly, mild autonomous cortisol secretion by adrenal adenomas is associated with an increased risk for hypertension and type 2 diabetes mellitus (17). Although associations do not prove cause and effect, these findings are concerning. Unfortunately, for logistical reasons it will be difficult, if not impossible, to perform multi-year, prospective, randomized trials to compare the outcome of conventional daily glucocorticoid replacement doses with lower more physiologic doses. Some valuable information may be derived from two short term trials comparing a lower prednisolone dose to the standard hydrocortisone dose (PRED-AID study, ISRCTN41325341 and HYPER-AID, NCT03608943).

## A pragmatic Approach to Individualizing the Daily Glucocorticoid Replacement Dose

If we accept that the daily glucocorticoid replacement dose range is broad, there is the potential for up to several fold overreplacement, if the current guidelines are applied to an individual of relatively low BSA and a pre-AI DCPR in the lower half of the normal range. It is generally agreed that laboratory measurements of serum plasma ACTH and serum cortisol concentrations and its surrogates are not useful for gauging the correct daily glucocorticoid replacement dose (1, 2). However, these measurements may serve a role in occasional patients that seem to have unusual HC requirements (18).

Consider two opposing approaches to determine the correct daily glucocorticoid replacement dose for an individual. The first approach is to treat with HC doses near the upper normal range (20 to 25 mg/d). Then lower the dose if the patient develops evidence of cortisol excess, such as central obesity, muscle weakness, violaceous abdominal striae, hypertension, diabetes, venous thrombosis, cardiovascular disease, myocardial infarction, stroke or osteoporosis. This approach has obvious disadvantages. These endpoints are delayed and either nonspecific or insensitive markers of mild glucocorticoid excess. Furthermore, irreversible end organ damage may develop before the glucocorticoid dose is decreased. A second approach is to slowly titrate to the lowest tolerated HC dose until there is evidence of mild AI, and then increase the dose slightly. The features of AI are expected to develop relatively rapidly, and be readily reversible. Since patients with AI can survive for many years undiagnosed (2), since mild under-replacement is unlikely to cause adrenal crisis in otherwise healthy individuals, and since many features of AI, such as weight loss and hypotension, are relatively sensitive and specific, then it is anticipated that this down titration approach will identify the correct daily glucocorticoid replacement dose for a given individual in a shorter time frame with potential for fewer adverse outcomes.

One retrospective study employed this down titration approach to empirically determine the daily glucocorticoid replacement dose in 25 otherwise healthy adults with either primary or secondary AI due to a variety of causes (19). Divided doses of HC or prednisone were chosen for the glucocorticoid replacement depending on the patient’s preference and drug availability. The prednisone to cortisol equivalence was assumed to be 4 to 1, a generally accepted ratio (20–22), although some suggest that it may be 7:1 (23). In each individual the glucocorticoid replacement was decreased by 5 mg/d HC or 1 mg/d prednisone every 2 to 6 months until one of the following predetermined AI end points precluded further titration: weight loss, symptomatic hypotension, hyponatremia, and otherwise unexplained fatigue. The titration was halted in 3 patients for hyponatremia, 22 patients for fatigue, and 0 patients for weight loss or symptomatic hypotension. For primary AI the mineralocorticoid dose was adjusted to normalize the plasma renin activity.

## Outcomes Using Daily Glucocorticoid Replacement Doses Below the Conventional Recommended Doses

The titration method of determining daily glucocorticoid replacement dose was evaluated for safety, and the final doses compared to both the current recommendations and to current estimates of DCPR (19). This empirically determined replacement dose did not carry an increased risk of adrenal crisis as compared to historical controls (19). The mean daily dose expressed in HC equivalents was 13.9 ± 6 mg, a value that is significantly lower than the currently recommended midpoint daily HC dose of 20 mg (p < 0.001) (19). The empirically determined daily dose range was approximately 7-fold (4 to 30 mg HC equivalents) in a diverse population and clinically distinguishable from the recommended 1.7-fold range (15 to 25 mg) (19). The mean daily glucocorticoid replacement dose in HC equivalents and corrected for BSA was 7.6 ± 3.4 mg/m^2^ (range 1.9-14.4 mg/m^2^). This was not significantly different from the reported mean DCPR of 7.0 mg/m^2^, and closely approximated the reported DCPR range (2.7-14 mg/m^2^) (19). There was no difference in the replacement dose for patients with primary or secondary AI.

In primary AI a 12 week crossover prospective trial reported that a delayed release HC caused small but significant decreases in weight, blood pressure, and glycated hemoglobin as compared to equal doses of three times daily HC. One possible explanation is that the bioavailability of the delayed release HC was about 80% of the standard oral HC dose (24).

In secondary AI, the association of daily glucocorticoid replacement dose and morbidity and mortality has been investigated. In patients with nonfunctional pituitary tumors (NFPT) patients and secondary AI, two studies report that HC equivalent doses ≥ 30 mg/d, as compared to <30 mg/d, were associated with a significantly increased death risk (25, 26) independent of other pituitary deficiencies. In a similar NFPT group, Hammarstrand, et al. reported that a HC equivalent dose > 20 mg/d was associated with significantly increased mortality (HR = 1.88, CI = 1.06 - 1.33), although this study did not account for other hormone deficiencies (27). Filipsson et al. found that hypopituitary patients with HC equivalent replacement ≥ 20 mg/d had an increased body mass index and lipid abnormalities, but no difference in HbA1C (16). These differences were present at baseline and after 1 year of growth hormone replacement, effectively excluding growth hormone deficiency as a confounding factor. Although associations do not prove cause and effect, these studies raise the concern that conventional glucocorticoid replacement is too high.

In secondary AI mixed results have been reported in prospective short duration studies that compare lower to conventional daily glucocorticoid replacement doses. In a 3-month trial, decreasing HC from 30 mg/d to 15 mg/d in 13 patients was well tolerated, but there was no change in a variety of cardiometabolic end points including blood pressure, weight, plasma and urinary electrolytes, and serum glucose or HbA1C (28). Different results were obtained by Danilowicz, et al, in a trial of 11 patients in whom the glucocorticoid replacement dose was reduced from 20-30 mg/d to 10-15 mg/d for 6 to 12 months. There was a significant reduction in weight, abdominal fat, lipid profile and improved quality of life (29). Petersons, et al. reversed the approach and increased HC dose from about 15 mg/d to 30 mg/d in a 7-day study. There was no change in fasting glucose or glucose tolerance (30). In a prospective crossover trial of 10 men comparing total HC replacement doses of 30 mg/d, 20 mg/d, and 15 mg/d for 6 weeks each, lower HC doses caused a significant improvement in bone turnover markers and arterial stiffness, but no change in blood pressure or insulin sensitivity (31, 32). The effects of excess glucocorticoids manifest slowly. Larger and longer duration studies with individualized lower glucocorticoid doses will be necessary to determine if the conventional glucocorticoid replacement doses are harmful.

## Potential Areas of Investigation

### Secondary AI

The appropriate diagnosis and glucocorticoid replacement of secondary AI is not addressed by the current Endocrine Society guidelines (1). In the review by Husebye, the glucocorticoid replacement recommendation is the same for both primary and secondary AI (2). It has been proposed that patients with partial secondary AI may require daily HC doses of 0 to 10 mg (33), and some studies have excluded secondary AI patients who do not require any daily glucocorticoid, so as not to bias results (19, 34). Future studies are necessary to define partial secondary AI more precisely and provide guidelines for daily glucocorticoid replacement.

### Individualizing the Daily Glucocorticoid Replacement Dose

The method of individualizing the daily glucocorticoid replacement dose requires further investigation. The current guidelines suggest using the lowest daily glucocorticoid replacement dose within the 15 mg/d to 25 mg/d range, but give no suggestions as to how this should be accomplished (1). In the titration study the individualized daily glucocorticoid replacement dose was determined by slowly decreasing the glucocorticoid by about 4 to 5 mg HC equivalent every 2 to 6 months to avoid confusing glucocorticoid withdrawal symptoms with AI symptoms and to avoid severe AI (19). The rate and timing of the titration method has not been validated by others.

There are special challenges to replacing glucocorticoids in patients with both primary AI and type 1 diabetes and in pregnant patients with AI. These are discussed elsewhere (35–37), and are subject to further investigation at lower replacement doses.

The current recommendations do not suggest adjustments for BSA and age. Patient populations have a large range of BSA, and the total daily cortisol production is BSA dependent. A significant positive association of DCPR with age has been reported (3), but the mechanism for this alteration and its physiologic importance are unknown. Investigations into adjustments for BSA and age are indicated.

### Glucocorticoid Replacement Timing and Alternate Glucocorticoids

As reviewed by others, methodologies such as continuous infusion, delayed absorption formulations, multiple small daily doses, and intermediate activity glucocorticoids have been studied at conventional replacement doses (11, 38). Using these methods to examine the outcome of a daily glucocorticoid replacement dose that is lower and more physiologic than current recommendations would be of interest.

The biologic equivalence and clinical utility of synthetic glucocorticoids require further investigation. This is particularly important for prednisolone and its hepatically activated precursor prednisone, that have widespread availability at a relatively low cost. A broad prednisolone/HC equivalence of 4 to 7 has been suggested (1, 22, 23). An observational study suggests a 6:1 equivalence, since AI subjects on an average of 3.7 mg/d prednisolone or 20.5 mg/d HC had equal cardiometabolic risk factors (39). A specific prednisolone assay may assist in determining equivalence and dosing schedules (39–41).

### The Normal DCPR May Be Too High in the Current Western Environment

We hypothesize that for some individuals their normal DCPR may be too high and harmful in the modern Western environment. Possibly the normal DCPR and its diurnal variation evolved as an orexigenic modifier to [1] drive appetite and food/salt seeking during the daylight hours, [2] to be low enough at night to allow for adequate sleep and recovery, and [3] modulate energy stores and help sustain blood pressure for 24 h. In the current Western environment, little activity is required to find, prepare and ingest adequate calories and salt to meet current modest energy requirements and maintain blood pressure. Within this environment the normal DCPR for some individuals may contribute to such morbidities as obesity, diabetes and hypertension. This may explain why some AI patients tolerate very low daily glucocorticoid replacement doses and why some patients with partial AI require no daily glucocorticoid replacement. Some extreme experiments of nature are also consistent with this hypothesis. Patients with hypothalamic obesity due to genetic disorders affecting hypothalamic MSH and ACTH production and patients with craniopharyngioma surgery affecting the hypothalamic satiety center have excessive appetites and weight gain even with AI (42, 43). Therefore, in the current environment, multiple orexigenic pathways may overcome the anorexigenic effects of glucocorticoid deficiency. The hypothesis could be tested using tools such as cortisol synthesis inhibitors and glucocorticoid receptor antagonists that are recently available.

## Summary and Conclusions

Our interpretation of the literature suggests that the current daily glucocorticoid replacement dose recommendations for otherwise healthy AI patients are based on assertions that do not account for the DCPR range, oral HC bioavailability of 100 percent, BSA diversity or age. The current midpoint recommended daily glucocorticoid replacement dose is about 30 percent greater than that predicted from the DCPR. Long-term therapy with conventional glucocorticoid replacement doses is associated with adverse outcomes, but cause and effect has not been established. A titration approach is safe and yields a daily glucocorticoid replacement dose that approximates the mean and range of the known DCPR.

## Author Contributions

The authors CC and CM contributed to the data extraction, data interpretation, and writing the manuscript.

## Conflict of Interest

The authors declare that the research was conducted in the absence of any commercial or financial relationships that could be construed as a potential conflict of interest.

## Publisher’s Note

All claims expressed in this article are solely those of the authors and do not necessarily represent those of their affiliated organizations, or those of the publisher, the editors and the reviewers. Any product that may be evaluated in this article, or claim that may be made by its manufacturer, is not guaranteed or endorsed by the publisher.
